# The Role of BMP Signaling in Endothelial Heterogeneity

**DOI:** 10.3389/fcell.2021.673396

**Published:** 2021-06-21

**Authors:** Orjin Han, Boryeong Pak, Suk-Won Jin

**Affiliations:** Cell Logistics Research Center, School of Life Sciences, Gwangju Institute of Science and Technology (GIST), Gwangju, South Korea

**Keywords:** heterogeneity, endothelium, BMP signaling, post-translational modification, computational modeling

## Abstract

Bone morphogenetic proteins (BMPs), which compose the largest group of the transforming growth factor-β (TGF-ß) superfamily, have been implied to play a crucial role in diverse physiological processes. The most intriguing feature of BMP signaling is that it elicits heterogeneous responses from cells with equivalent identity, thus permitting highly context-dependent signaling outcomes. In endothelial cells (ECs), which are increasingly perceived as a highly heterogeneous population of cells with respect to their morphology, function, as well as molecular characteristics, BMP signaling has shown to elicit diverse and often opposite effects, illustrating the innate complexity of signaling responses. In this review, we provide a concise yet comprehensive overview of how outcomes of BMP signaling are modulated in a context-dependent manner with an emphasis on the underlying molecular mechanisms and summarize how these regulations of the BMP signaling promote endothelial heterogeneity.

## Introduction

Endothelial cells (ECs) exhibit great heterogeneity (Aird, 2003), in their developmental origin (Bautch and Caron, 2015; Pak et al., 2020), morphology (Sailem and Al Haj Zen, 2020), as well as gene expression (Chi et al., 2003). With technical advances, the concept of endothelial heterogeneity has been re-evaluated and extended at the single-cell level (Nolan et al., 2013; Kalucka et al., 2020), which re-ignited the interest in signaling pathways essential for modulating endothelial heterogeneity in living organisms. In order to contribute to endothelial heterogeneity, signaling pathways should satisfy certain criteria. For instance, signaling pathways that enrich endothelial heterogeneity should not provide essential functions for endothelial survival but rather function as “auxiliary” cues. In addition, such signaling should elicit its effects in a context-dependent manner. Therefore, signaling pathways, which provide non-essential roles and have been shown to elicit angiogenic responses from a selected subtype of ECs without affecting the viability of ECs, are likely to be instrumental in creating endothelial heterogeneity. Conversely, signaling pathways such as vascular endothelial growth factor-A (VEGF-A) signaling, which are indispensable for the survival of ECs and elicit robust responses from all subtypes of ECs (Byrne et al., 2005), are less likely to contribute to endothelial heterogeneity.

**FIGURE 1:**
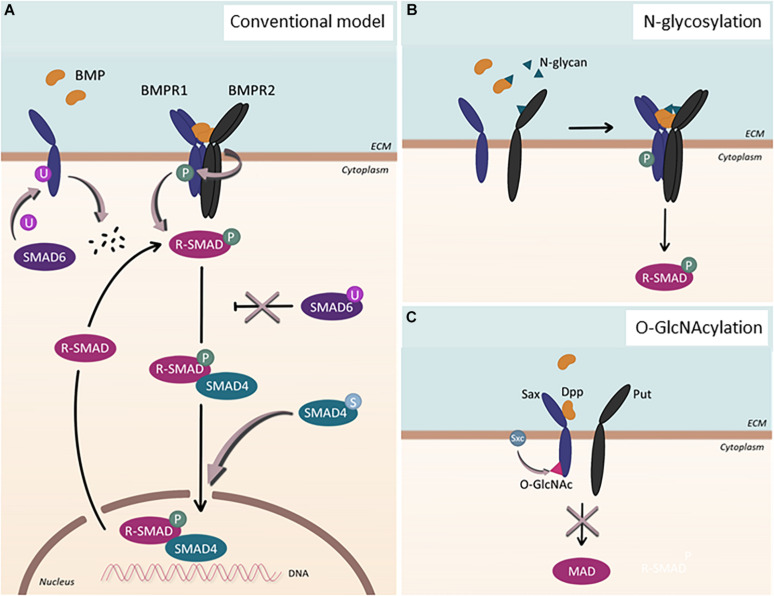
Post-translational modification of BMP signaling components. **(A)** Conventional model of BMP signaling pathway. Upon BMP ligand binding, BMP type 1 receptor (BMPR1) is phosphorylated. Subsequently, R-SMADs, SMAD1/5/8, become phosphorylated and, together with SMAD4, localize into the nucleus to regulate gene transcription. This process can be inhibited by SMAD6. Additional post-translational modifications affect the signaling pathway in various ways: SMAD6 can regulate BMPR1 by facilitating its ubiquitination and subsequent degradation. Upon ubiquitination of SMAD6, SMAD6 loses its ability to inhibit the BMP signaling cascade, which ultimately leads to upregulation of the signaling pathway. When SMAD4 undergoes sumoylation, nuclear accumulation of the protein is enhanced. **(B)** N-glycosylation. N-glycans can bind to BMP ligands as well as to BMP type 2 receptors (BMPR2), enabling proper folding and binding of the proteins. **(C)** O-GlcNAcylation. In *Drosophila*, Sxc facilitates O-GlcNAcylation of Sax, the ortholog for human ALK2, resulting in the attenuation of the signaling pathway.

As one of the archetypal receptor-ligand-mediated signaling pathways, bone morphogenetic protein (BMP) signaling has gained increasing importance in the development and homeostasis of the vascular system. Its ability to selectively activate ECs in a context-dependent manner without influencing the survival of ECs (Cunha et al., 2017; Goumans et al., 2018) satisfies the aforementioned criteria of a signaling pathway of which function is important for establishing and promoting endothelial heterogeneity. Belonging to the transforming growth factor-β (TGF-β) family, BMP signaling forms the largest subgroup with more than a dozen ligands and four BMP-specific type I (Miyazono et al., 2010). The canonical signaling pathway is initiated by a dimeric ligand binding to its heterotetrameric receptor complex, consisting of two type I and two type II receptors, leading to the phosphorylation of the receptors (Figure 1A). The activated receptors, in turn, phosphorylate downstream target SMAD1/5/8, which translocates into the nucleus in association with SMAD4 to modify transcriptomic profiles. In addition, it has been shown that BMP signaling could activate mitogen-activated protein kinase (MAPK) pathway in a context-dependent manner (Massague, 2003).

To better understand how BMP signaling modulates endothelial heterogeneity, it is essential to understand how BMP signaling functions within ECs. However, a simplified schematic diagram fails to capture the complex molecular landscape of BMP signaling, since distinct expression patterns of individual BMP signaling components result in diverse context-dependent outcomes (Seeherman et al., 2019). In addition, certain combinations of ligands and receptors differently regulate signaling, generating diverse cellular responses (Antebi et al., 2017). Moreover, numerous factors modulate the amplitude and duration of BMP signaling to further increase the complexity of BMP signaling. In this review, we provide a general overview of how BMP signaling functions in ECs and contributes to endothelial heterogeneity. We summarize how distinct responses toward BMP stimulation within subtypes of ECs contribute to endothelial heterogeneity.

## Endothelial Heterogeneity and BMP Signaling

Bone morphogenetic protein signaling appears to function as a critical factor that enhances endothelial heterogeneity. During embryogenesis, it has been shown that BMP signaling promotes the specification of venous ECs (Neal et al., 2019), therefore, sets apart arterial and venous fates, and modulates the initial setup of endothelial heterogeneity. Lack of *Alk3* within naïve ECs led to the failure of venous specific marker expression in presumptive venous ECs (Neal et al., 2019). In sprouting angiogenesis, BMP signaling has been shown to modulate dynamics between tip and stalk cell fates during sprouting angiogenesis (Moya et al., 2012), thereby enriching the endothelial heterogeneity.

Based on the subtypes, cellular responses toward BMP stimulation appear to be distinct among ECs which could further reinforce the endothelial heterogeneity. For instance, BMP signaling elicits distinct outcomes in blood ECs (BECs) and lymphatic ECs (LECs), the two major subtypes of ECs (Wiley and Jin, 2011; Coso et al., 2014). Therefore, it appears as if the responses toward BMP ligands in LECs are opposite to those in BECs. Given that the majority of LECs emerge via transdifferentiation of BECs, primarily venous in nature (Oliver and Srinivasan, 2010), it appears that the propensity to respond to a specific BMP ligand alters as a part of the transdifferentiation program. While it is apparent that BECs and LECs distinctively respond to BMP stimulation, it is largely unknown which factors are responsible for creating subtype-specific responses toward BMP stimulation. Considering this built-in redundancy of BMP signaling at the level of ligands, receptors, as well as effectors, it is tempting to speculate that these two subtypes of ECs may utilize pre-dominant receptors and effectors, which could facilitate subtype-specific responses. Recent analyses on the expression of BMP signaling components corroborate this idea (Yoshimatsu et al., 2013; Takeda et al., 2019).

Even within BECs, it has been shown that the responsiveness toward BMP signaling also varies between arterial and venous ECs. For instance, we and others have shown that pro-angiogenic BMP signaling could selectively induce angiogenic responses in venous ECs without affecting arterial ECs (Wiley et al., 2011; Kashiwada et al., 2015). Together, these studies highlight the innate differences of arterial and venous ECs in BMP responsiveness, which appears to modulate sprouting angiogenesis, potentially in conjunction with Notch and VEGF-A signaling. For instance, Notch signaling has been shown to interact with BMP signaling to orchestrate the intricate process of sprouting angiogenesis (Moya et al., 2012). Moreover, Notch renders the responsiveness of ECs toward pro-angiogenic BMP stimuli, BMP2 and BMP6, by regulating the expression of SMAD6, which ultimately contributes to the phosphorylation of SMAD1/5 and a tip cell phenotype (Mouillesseaux et al., 2016). Recent findings also implicated synergistic effects of BMP signaling and VEGF signaling on tip cell-associated markers VEGFR2 and DLL4 (Pulkkinen et al., 2020). Furthermore, Notch signaling also appears to influence BMP signaling at the effector level since the expression of Herp2 in ECs is co-modulated by Notch and BMP6 signaling (Itoh et al., 2004). Taken together, these studies reiterate the significance of BMP signaling and its mutual interaction with other signaling pathways in regulating endothelial behavior during angiogenesis.

## Factors That Modify Cellular Outcomes of BMP Signaling

Bone morphogenetic protein signaling seems to be highly context-dependent, eliciting opposite outcomes in similar cell types (Abe et al., 2000; Shu et al., 2011). It appears that multiple layers of modulation collectively shape the signaling landscape of BMP ligands: Ligand–receptor interaction in diverse combinations which can be further modulated by agonists and antagonists, mechanical forces which are sensed by the cell and transmitted into biochemical cues, and post-transcriptional and post-translational regulations which provide fine-tuning for the signaling pathway, collectively formulate outcomes of BMP signaling with a remarkable degree of flexibility and versatility. Only now, we begin to understand the influence of these factors in BMP signaling.

### Promiscuity of Ligand–Receptor Interaction

As in other signaling cascades, BMP signaling features a multitude of ligands and receptors, which associate and communicate with each other in diverse ways. However, the multiplicity of these components is not employed equally; most of them exhibit redundant functions. Previous works have shown that a multitude of BMP ligands binds to the same receptor (Mueller and Nickel, 2012) with different affinities (Seeherman et al., 2019). However, there are practical limitations in determining the ligand–receptor promiscuity in living organisms. To circumvent these hurdles, Antebi et al. (2017) resorted to mathematical modeling. By elegantly demonstrating the ligand–receptor promiscuity and the complex reciprocities between its signaling components, they suggested that the redundancy of BMP signaling components might manifest specific signaling-processing capabilities. While this needs to be validated *in vivo*, computational analyses revealed that cells distinguish distinct signaling environments, such as the concentration of ligands and differences in ligand–receptor complexes, to respond to BMP stimulation. Interestingly, inside the signaling-receiving cells, the canonical downstream target SMAD1/5/8 is activated regardless of the signaling-conferring ligand–receptor complex, suggesting the possibility that signaling-receiving cells are only capable of perceiving the sum of BMP ligand stimulation (David and Massague, 2018). This scenario, which is feasible in theory, certainly contradicts the prevailing idea that each BMP ligand retains its unique role *in vivo* (Wang et al., 2014). Therefore, further analyses are warranted to fully understand how the promiscuity of ligand–receptor interaction affects the context-dependent and heterogeneous signaling outcomes of BMP signaling.

### Environmental Factors

It has been shown that mechanical forces affect the outcomes of BMP signaling in a number of cell types (Sedlmeier and Sleeman, 2017). For instance, abrogation of α5β1 integrin in vertebrate embryos substantially decreases the response of chondrocytes toward BMP stimulation (Garciadiego-Cazares et al., 2004, 2015). ECs provide another example of responsiveness toward BMP stimulation which is modulated by environmental factors. Previously, shear stress, the mechanical force created by laminar flow, has been implicated in regulating BMP signaling in ECs (Min and Schwartz, 2019; Hiepen et al., 2020). Upon exposure to shear stress, SMAD1/5/8 becomes phosphorylated in both BECs and LECs, dependent on the type and magnitude of shear stress. While turbulent shear stress induced by low oscillatory flow promotes EC proliferation and leads to SMAD1/5/8 phosphorylation (Zhou et al., 2012), laminar shear stress contributes to the quiescence of ECs through the BMP9-ALK1/ENG-SMAD1/5/8 axis (Baeyens et al., 2016), potentially going through the primary cilia (Vion et al., 2018). Interestingly, SMAD1 was most strongly affected by laminar shear stress, whereas SMAD5 and SMAD8 were less responsive (Baeyens et al., 2016). Moreover, the depletion of SMAD4 in human coronary artery ECs impaired the alignment of ECs to blood flow and led to an increased diameter of coronary arteries in developing mice (Poduri et al., 2017). Therefore, it appears that the types of flow, potentially modulating the activity of distinct SMADs, influence outcomes of BMP signaling in ECs. In addition to mechanical forces, recent studies reported hypoxia as one of the potential modulators for BMP signaling. Under hypoxic conditions, differential expression of BMP ligands could be observed in comparison to normoxic conditions (Pulkkinen et al., 2021). Moreover, BMP signaling could also attenuate hypoxic responses in ECs through the ALK1/SMAD/ATOH8 axis, ultimately impeding the development of pulmonary arterial hypertension (PAH) (Morikawa et al., 2019).

### Transcriptional and Post-transcriptional Regulation

The responses toward BMP stimulation are further regulated by modifying BMP signaling components. For instance, the transcriptional efficacy of BMP signaling target genes can be modulated by epigenetic factors and other transcriptional co-factors. SMAD1 interacts with p300/CREB-binding protein (CBP) *in vitro* and *in vivo*, which enhances upon phosphorylation of SMAD1 (Nakashima et al., 1999; Pearson et al., 1999). In addition, SMAD1 can form a ternary complex with Notch intracellular domain in the presence of p300/CBP and P/CAF to augment transcriptional activation of Notch target genes (Takizawa et al., 2003). Moreover, chromatin immunoprecipitation analysis in ECs and pulmonary arterial smooth muscle cells revealed that SMAD1/5 preferentially binds to the GC-rich SMAD binding element outside the promoter of known genes, which appears to be mediated by epigenetic factors (Morikawa et al., 2011). Furthermore, responses toward BMP signaling are modulated by post-transcriptional regulation. For instance, numerous miRNAs are involved in BMP signaling-regulated processes (Hata and Kang, 2015). Recent studies provided persuasive evidence of the role of miRNAs in modulating BMP signaling (Dunworth et al., 2014). Therefore, it is apparent that transcriptional as well as post-transcriptional regulation of BMP signaling components could alter the landscape of BMP signaling.

### Post-translational Regulation

Post-translational modifications (PTMs) have been shown to contribute to the extensive versatility and complexity of signaling pathways in eukaryotic cells. Perturbations of PTMs have been linked to various diseases, illustrating the significance of PTMs in maintaining cellular homeostasis (Li et al., 2010; Karve and Cheema, 2011). While PTMs in the TGF-β signaling pathway (Xu et al., 2012) have been studied intensively, far less is known about them in BMP signaling. The best-studied PTM in the BMP signaling is phosphorylation, the reversible attachment of a phosphoryl group to its receptors and downstream targets (Figure 1A). Upon ligand binding, successive phosphorylation ensues (Nickel and Mueller, 2019); the constitutively active BMPR2 phosphorylates the GS-domain of BMPR1s (Wrana et al., 1994), which, in turn, phosphorylate the SSXS motif of SMAD1/5/8 (Chen et al., 1997; Kretzschmar et al., 1997; Suzuki et al., 1997).

In addition to phosphorylation, ubiquitination and sumoylation, both of which are reversible PTM, have been shown to modulate the context of BMP signaling (Hershko and Ciechanover, 1998) (Figure 1A). The primary target of ubiquitination within BMP signaling appears to be SMAD1/5 (Zhu et al., 1999), though other components of BMP signaling, including SMAD6 and ACVR1/ALK2, could also undergo ubiquitination (Zhang et al., 2013; Herhaus et al., 2014; Seo et al., 2019). In addition, several studies have shown that SMAD4 could be sumoylated, which promotes SMAD4 accumulation into the nucleus and prolongs BMP signaling (Lin et al., 2003). Considering the complex interplay between ubiquitination and sumoylation (Liebelt and Vertegaal, 2016), balance between these PTMs may provide additional regulatory input for BMP signaling, which, however, requires further investigation.

Beyond these well-studied PTMs, other less established protein modifications have been acknowledged to be of great significance in modulating BMP signaling outcomes, including methylation, acetylation, O-GlcNAcylation, and acylation (Figures 1B,C). For instance, O-GlcNAcylation, which is the addition of a single *O*-linked *N*-acetyl-glucosamine (*O*-GlcNAc) moiety to target proteins, has been shown to fine-tune *Drosophila* BMP type I receptor Saxophone (Sax), an ortholog for human ALK2 (Moulton et al., 2020). In addition, it has been reported that a number of BMP ligands and BMPR2 undergo N-linked glycosylation, the addition of glycan to the target proteins (Garrigue-Antar et al., 2002; Hang et al., 2014; Negreiros et al., 2018). Interestingly, the N-glycosylation consensus of BMPR2 was positioned at a site that is mutated in heritable PAH patients (Pfarr et al., 2011; Lowery et al., 2014), alluding to the importance of this PTM for modulating the activity of BMP signaling. Increasing evidence also suggests the regulation of BMP signaling by methylation by which the inhibitory effect of Smad6 could be ensured (Xu et al., 2013; Wu et al., 2021).

## The Role of BMP Signaling in Endothelial Dysfunction

**FIGURE 2:**
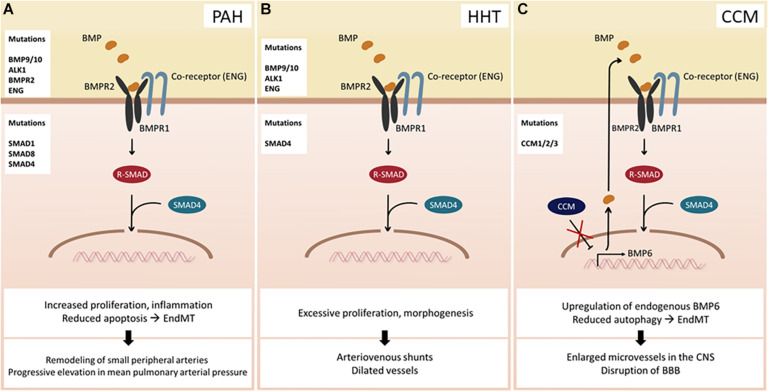
Consequences of BMP signaling dysfunction. **(A)** Pulmonary arterial hypertension (PAH) can occur due to mutations in BMP ligands, receptors, co-receptors, and SMAD proteins. **(B)** Hereditary hemorrhagic telangiectasia (HHT) arises upon mutation of BMP9/10, ALK1, ENG, or SMAD4. **(C)** Cerebral cavernous malformation (CCM) develops when one of the CCM proteins is mutated. Hence, transcriptional activity of BMP6 increases leading to an enhanced BMP signaling. *Abbreviations*: CNS, central nervous system; BBB, blood–brain barrier.

Numerous studies have suggested that dysregulation of BMP signaling in ECs could lead to various diseases. For instance, mutations in BMPR2 predispose to the onset of PAH (Figure 2A), a rare chronic disease characterized by an increase in mean pulmonary arterial pressure, which eventually leads to failure of the right ventricle (Dunmore et al., 2021). Recent studies using next-generation sequencing (NGS) technology have identified more than 800 mutations, including 486 distinct, non-recurrent variants, many of which affect BMP signaling in ECs (Southgate et al., 2020). Moreover, other BMP-related genes have also been implicated with PAH (Graf et al., 2018). Another well-established disease associated with dysfunctional BMP signaling in ECs is hereditary hemorrhagic telangiectasia (HHT) (Figure 2B) and cerebral cavernous malformation (CCM) (Figure 2C), both of which are autosomal dominant disorders. The clinical hallmarks of HHT are dilated vessels and arteriovenous shunts, which are prone to rupture. To date, mutations in a number of BMP signaling components, including Endoglin, ALK1, SMAD4, and more recently, BMP9 (McAllister et al., 1994; Johnson et al., 1996), have been identified in a smaller group of patients. However, the underlying molecular basis of HHT has not been fully elucidated. CCM is another hereditary condition that leads to enlarged microvessels in the central nervous system, which are susceptible to hemorrhage due to loss-of-function mutations in one of the three autosomal genes collectively known as the *CCM*s (Labauge et al., 2007). Deletion of any of the three genes results in increased synthesis of endogenous BMP6, which, in part, is promoted by KLF4 (Cuttano et al., 2016). Moreover, endothelial-specific deletion of *Ccm1* and *Ccm3* in mice induces endothelial to mesenchymal transition (EndMT), which contributes to the development of vascular lesions (Maddaluno et al., 2013; Malinverno et al., 2019), hinting that dysregulation of BMP signaling plays a key role in the progression of the CCMs. It is worth noting that only a subset of ECs appears to be affected by the pathological conditions caused by dysregulation of BMP signaling. For instance, arteriovenous shunts formed in HHT patients are initiated by a subset of venous ECs, which undergo excessive proliferation and morphogenesis (Brinjikji et al., 2015). In addition, while PAH promotes EndMT, only a subset of ECs in PAH patients transforms into vascular smooth muscle cells (Ranchoux et al., 2015). Therefore, it is fathomable that the effects of dysregulated BMP signaling on ECs are also highly heterogeneous, reminiscent of normal physiological responses toward BMP signaling.

## Perspectives

As we obtain comprehensive knowledge on the role of BMP signaling in endothelial heterogeneity, we are confronted with a plethora of enigmas. Increasing evidence indicates that the complexity of BMP signaling dynamics provides essential regulation on endothelial plasticity. Emerging reports have identified novel fine-tuning regulatory mechanisms for BMP signaling. While their functions remain largely unknown in the endothelium, given the ubiquitous expression of these regulators, it is plausible that BMP signaling in ECs is similarly modulated. Since the role of BMP signaling in endothelial heterogeneity is still in its infancy, it will be of great importance to elucidate distinct regulatory mechanisms and more importantly its impact on the endothelial phenotype, which ultimately will lead to distinct physiological responses in the body. The plethora of regulatory possibilities provide an additional dimension for regulating distinct BMP signaling components and therefore further enrich the impact of the BMP signaling on endothelial heterogeneity. Not yet identified environmental factors, as well as various PTMs, might give us answers to yet unsolved enigmas and offer novel strategies to overcome limitations faced so far. Therefore, further investigations will provide insights into how such a conceptually simple signaling cascade can give rise to exquisite plasticity and versatility. Moreover, the availability of novel techniques, such as NGS, single-cell analysis, and bioinformatics, will refine our understanding of endothelial dysfunctions and concomitant diseases and ultimately contribute to the development of new therapeutic interventions for various diseases by fully harnessing the heterogeneity and context-dependent properties of BMP signaling.

## Author Contributions

All authors wrote and edited the manuscript.

## Conflict of Interest

The authors declare that the research was conducted in the absence of any commercial or financial relationships that could be construed as a potential conflict of interest.
